# Child Nutrition Trends Over the Past Two Decades and Challenges for Achieving Nutrition SDGs and National Targets in China

**DOI:** 10.3390/ijerph17041129

**Published:** 2020-02-11

**Authors:** Bo Yang, Xin Huang, Qin Liu, Shenglan Tang, Mary Story, Yiwen Chen, Maigeng Zhou

**Affiliations:** 1 School of Public Health and Management, Research Center for Medicine and Social Development, Collaborative Innovation Center of Social Risks Governance in Health, Chongqing Medical University, Chongqing 400016, China; 2017111053@stu.cqmu.edu.cn (B.Y.); 2017111052@stu.cqmu.edu.cn (X.H.); 2016111061@stu.cqmu.edu.cn (Y.C.); 2 Department of Population Health Science and Duke Global Health Institute, Duke University, Durham, NC 27708, USA; shenglan.tang@duke.edu; 3 Duke Global Health Institute, Duke Kunshan University, Kunshan, Jiangsu 215316, China; 4 Department of Family Medicine and Community Health and Duke Global Health Institute, Duke University, Durham, NC 27708, USA; mary.story@duke.edu; 5 National Center for Chronic and Noncommunicable Disease Control and Prevention, Chinese Center for Disease Control and Prevention, Xicheng District, Beijing 100050, China; maigengzhou@126.com

**Keywords:** Nutrition, Malnutrition, SDGs, China’s national plans, Children

## Abstract

Background: The objectives of the current study were to identify the trends in child nutrition, the gaps in achieving child nutrition-related goals, and implications for program and policy options for the Chinese government. Methods: Eight child nutrition-related indicators from the Sustainable Development Goals (SDGs) and China’s national nutrition plans, and two datasets, Global Burden of Disease 2016 and Chinese National Nutrition and Health Surveillance, were used in our analysis. Results: Over the past 26 years, the prevalence of stunting, wasting, and underweight for children under 5 years was reduced by 58.7%, 53.4%, and 69.2%, respectively. Overweight for children aged 1–4 years increased 88.9% and obesity increased 2.14 times. Exclusive breastfeeding of newborns (7–28 days) was stable, at about 30%. We estimated child wasting would be 3.0% lower than the target of 5.0% based on predictive values for meeting the SDGs in 2025. The number of stunted children under five years would be reduced by 39.7%, while overweight would increase 2.2% throughout China. Conclusion: These results highlight the urgent need for targeted policies and interventions to reduce child stunting and overweight and increase exclusive breastfeeding to improve child health and meet the SDG targets and China’s national goals.

## 1. Introduction

Nutrition has profound impacts on health throughout the life course and it is inextricably linked with cognitive and social development, especially in early childhood [1]. Economic or social resources are linked to malnutrition, which encompasses both undernutrition and overweight/obesity. An estimated 45% of deaths of children under age five in low and middle income countries are linked to malnutrition [1]. Malnutrition also impedes children’s achievement of their full economic, social, educational, and occupational potential. Further, overweight and obesity may result in premature mortality and the early onset of chronic diseases, such as diabetes and cardiovascular diseases with high levels of disability [2]. Therefore, malnutrition, in all its forms, is a global public health issue.

Great gains have been made in child nutrition during the Millennium Development Goals (MDGs, 2000–2015); however, globally there still are 159 million children under age five with stunting, 50 million children under age five with wasting, and 41 million children are overweight in 2015 [3]. Among infants aged 0–5 months, 61% are not exclusively breastfed [4]. The number of overweight children under five in low-income countries has nearly quadrupled since 1990 [5]. Therefore, the United Nations (UN) released Sustainable Development Goals (SDGs) in 2015 for the year 2030. Of the 17 SDG goals, SDG 2.2 focuses on improving nutrition and ending all forms of malnutrition, including achieving by 2025, the internationally agreed targets on stunting and wasting in children under five years of age and addressing the nutritional needs of adolescent girls, pregnant and lactating women, and older persons [6].

Remarkable progress was made in reducing child undernutrition in China during the MDGs. The rate of stunting and underweight in children under six years has fallen by half from 2002 to 2013, the prevalence of overweight increased from 6.5% to 8.4% during the same period [7]. However, sex and regional disparities exist in child malnutrition. The prevalence of stunting is higher for boys than girls [7], and 1 out of 10 children under five years is stunted in the central and western parts of China. The Chinese government has implemented several nutrition targets to improve child nutrition [8], including the National Program for the Development of Children (2011–2020) [9] and the National Nutrition Program (2017–2030) [10], to meet the nutrition-related SDGs in 2025.

We investigate the status and trend of child nutrition in china over past two decades, and predicted values of these indicators by the target year, including sex and region disparities and analysis of each indicator to identify the priority nutrition areas China needs to focus on, to support the achievement of these nutrition related targets in China.

## 2. Materials and Methods

### 2.1. Source of Data

The study used data from the Global Burden of Disease (GBD) 2016, for six data points (year 1990, 1995, 2000, 2005, 2010, and 2016). GBD is an approach to global descriptive epidemiology of more than 350 diseases and injuries and exposure risk factors allowing for comparisons over time, across age groups, and among populations, which were developed by the Institute for Health Metrics and Evaluation (IHME) at the University of Washington [11]. However, because two indicators, prevalence of low birthweight and exclusive breastfeeding (EBF) for Chinese infants under six months, were not included in GBD 2016, we added the database of the Chinese National Nutrition and Health Surveillance (CNNHS), in which two data points (2002 and 2013) were used. CNNHS is a national nutrition monitoring survey conducted every 3–4 years to understand the nutritional status and risk factors among Chinese citizens [12]. It covers 30 provinces, municipalities, and autonomous regions (excluding Taiwan, Hong Kong, and Macao), with a sample size of 34,650 children aged 0–5 years in 2013 [7]. The details of the GBD study [13] and CNNHS [14] have been reported elsewhere.

### 2.2. Child Nutrition-Related Indicators

We selected the child nutrition-related indicators from the SDG goals and China’s national nutrition plans. The SDG goals contain five nutrition-related indicators [15]: (1) 40% reduction in children younger than five years who are stunted, (2) reduce and maintain childhood wasting to less than 5%, (3) no increase in childhood overweight, (4) increase rate of exclusive breastfeeding until six months postpartum to at least 50%, and (5) 30% reduction in the annual incidence of low birthweight.

China’s national nutrition plans included another three targets: To reduce the prevalence of (1) stunting to less than 7% and 5% in 2020 and 2030, (2) underweight to less than 5% in 2020, and (3) low birthweight to less than 4% in 2020. All if the indicators in the SDG goals and China’s national nutrition plans were targeted to children under five years and infants under six months. From the two databases, eight indicators were included in this study. The estimated prevalence of child stunting, wasting, underweight under five years, overweight, and obesity for children aged 1–4 years, and EBF for infants aged 7–28 days at national, sex, and regional levels were obtained from the GBD 2016. The estimates prevalence of EBF up to six months and low birthweight was obtained from CNNHS.

### 2.3. Statistical Analysis

We describe child nutrition status and the percentage point changes from 1990 to 2016, and changes of EBF under six months and low birthweight from 2002 to 2013 at national, sex, and regional levels. The regional disparities were based on official statistical categorization of eastern, central, and western parts of China in line with the socio-economic development status [16]. Rural and urban disparities were also explored in the prevalence of EBF in infants, when data were available.

We projected the specific absolute values for nutrition indicators at the national, sex, and regional levels by the modified projection model Trend Calculator, derived from the GBD 2016 SDG study [13], and then compared the projective values with the SDGs and national targets to identify the prioritized key areas. The low birthweight indicator was not included in the projections, because of insufficient data points. We only compared the status of low birthweight with China’s national plan in 2020. See Supplementary Materials for Trend Calculator.

The study was conducted in accordance with the Declaration of Helsinki, and The Ethics Committee of Duke University approved the protocol (2017-1359).

## 3. Results

### 3.1. Status, Trends and Sex/regional Disparities of Child Nutrition Indicators

#### 3.1.1. Undernutrition of Children Under 5 Years Old

Over a 26 year period from 1990–2016, for children under five years old, the prevalence of child stunting was reduced by 58.7% (from 28.1% to 11.6%), wasting was reduced by 53.4% (from 5.8% to 2.7%), and underweight was reduced by 69.2% (from 12.0% to 3.7%) (Table 1, Figure S1).

Sex and regional disparities were found. In 2016, the stunting and wasting rates were 1.2 times higher among boys, while the underweight rate was 1.05 times higher among girls. Stunting, wasting, and underweight rates were 1.47, 1.50, and 1.96 times higher in western China, as compared with eastern China. From 1990 to 2016, the decline of stunting, wasting and underweight rates in western China (18.6%, 3.7% and 10.2%) and in girls (17.2%, 3.4% and 8.4%) was higher than those in other two regions and boys (Figure S2).

#### 3.1.2. Overweight and Obesity of Children Aged 1–4 Years Old

Among children aged 1–4 years old from 1990 to 2016, the prevalence of overweight increased by 88.9% (from 6.3% to 11.9%), and obesity increased 2.14 times than that in 1990 (from 2.2% to 6.9%) (Table 1, Figure S3).

Sex and regional disparities also exist and gradually widened over the past 26 years. The overweight and obesity rates were 1.45 times and 1.50 times higher among boys, and 1.43 and 1.55 times higher in eastern China when compared to the lowest rates in western China. Eastern China had the greatest increase (6.3% overweight and 5.2% obesity) over time when compared to other regions. Boys had higher rates of child overweight (5.6%) and obesity (5.4%) as compared to girls (5.2% and 3.9%) (Figure S4).

#### 3.1.3. EBF of Infants under 6 Months and Newborns Aged 7–28 days

EBF of newborns (7–28 days) has been relatively stable in China over the past 26 years: 30.0% in 1990 and 30.2% in 2016 (Table 1). The EBF rate in central China and western China increased by 1.86% and 2.07%, but decreased by 1.19% in eastern China. Regional disparities exist; in 2016 the EBF rate was 1.2 times higher among western China, when compared with eastern China, which had the lowest EBF rate (27.3%). Regional disparities also increased from 1990 to 2016 (Figure S5). The overall EBF prevalence for infants aged six months and younger in China was 20.8% and 19.6% in urban areas and 22.3% in rural areas in 2013.

#### 3.1.4. Low Birthweight

Low birthweight prevalence in China from 2002–2013 was slightly reduced from 3.6% to 3.3%. Sex and urban-rural disparities were observed. Low birthweight was 1.13 times higher in girls and 1.36 times higher in urban areas in 2013.

### 3.2. Gaps in Achieving Nutrition-Related SDGs and National Nutrition Targets in China

**Figure 1:**
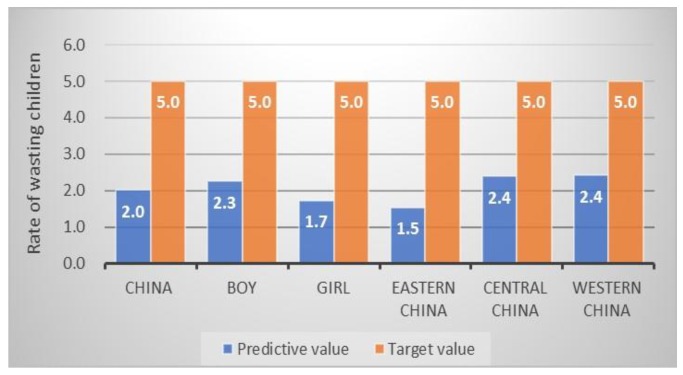
The estimated rate of Chinese wasting children and SDG target in 2025 (%).

**Figure 2:**
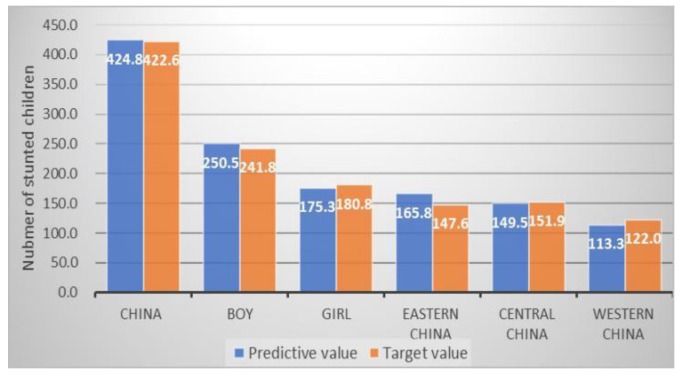
The estimated number of Chinese stunted children and SDG target in 2025.

Among the SDG targets (Table 2), reducing wasting in children under age 5 has been achieved in China, which is 3.0% and lower than the 5.0% of SDG target (Figure 1). Based on our predictive estimates, the number of children with stunting under five years would be reduced by 39.7% throughout China and reduced by 37.9% in boys, 41.8% in girls and 32.6%, 40.9%, 44.3% in eastern, central, and western China, respectively. Therefore, we would achieve the SDGs target of 40% reduction (Figure 2).

**Figure 3:**
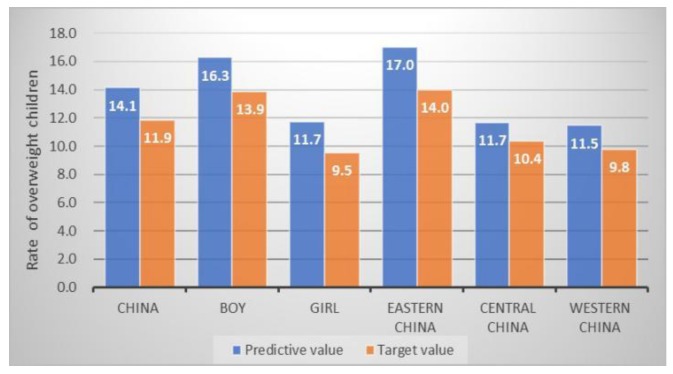
The estimated rate of Chinese overweight children and SDG target in 2025 (%).

**Figure 4:**
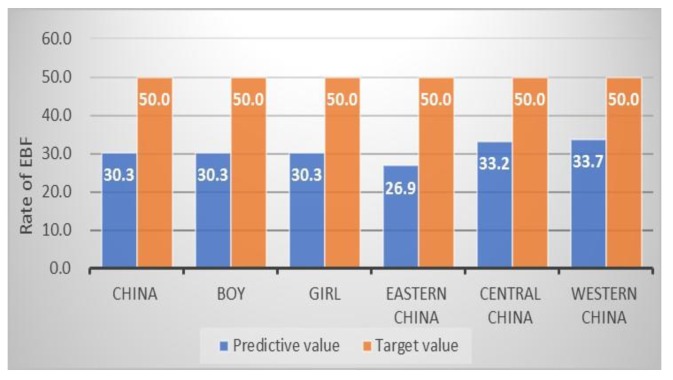
The estimated exclusive breastfeeding (EBF) rate of Chinese children and SDG target in 2025 (%).

For child overweight and EBF, there would be a large gap in meeting the SDG targets in 2025. The prevalence of child overweight would increase 2.2% throughout China and it is higher than the zero-increase target goal (Figure 3). The estimates of overweight among boys (2.4%) and eastern China (3.0%) would have the largest increase, when compared with girls and other two regions. Additionally, we estimate that the prevalence of EBF in infants aged 7–28 days would be 30.3% in China in 2025, which is 19.7% lower than the target set nationally for the three regions. The prevalence in eastern China is 23.1% lower than the SDG target (Figure 4).

**Figure 5:**
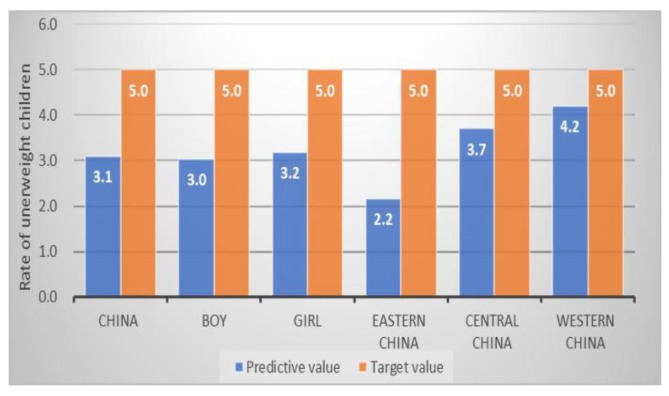
The estimated rate of Chinese underweight children and national nutrition target in 2020 (%).

**Figure 6:**
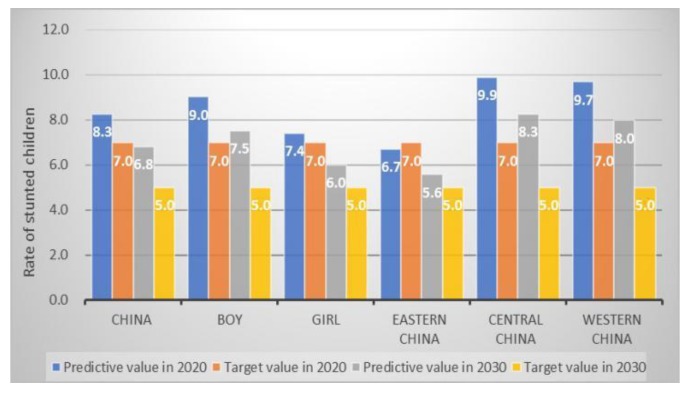
The estimated rate of Chinese stunting children and national nutrition target (%).

When compared with national targets, we found the target for low birthweight has already been achieved in 2016, with a prevalence of 3.3%. Child underweight is also in good standing, both at the regional level and for boys and girls. The prevalence estimates for underweight is 3.1% in 2020, which is 1.9% lower than the target value that was set in China’s National Plan for 2020 (Figure 5). Regarding child stunting rate in China, we found that only eastern China could achieve the target of 7.0% in 2020, with an estimated prevalence of 6.7%. Therefore, China as a country would not be able to achieve the stunting target by 2030 set in the national nutrition plan 2017–2030 (Figure 6).

## 4. Discussion

This is the first study to analyze two large data sets to the best of our knowledge, the GBD 2016 and CNNHS to examine child nutrition status and secular trends over the past two decades in China, and predict values of key nutrition indicators at the national, sex, and regional levels, in order to meet target goals. First, we found that over the past 26 years, undernutrition in children under five years in China and low birthweight has remarkably improved, while child stunting, while improved, is still high, at 11.6%. Of serious public health concern, the prevalence of child overweight and obesity is progressively increasing in China, even among lower income regions. The low prevalence of infant EBF up to six months is also of concern. Second, sex and regional disparities exist in all the malnutrition indicators. Third, large gaps exist for meeting child stunting, overweight, and EBF goals, especially for child overweight and EBF, when compared to predicted values for target years and targets set for the SDGs and the Chinese government.

Our findings are consistent with data from the National Survey on Physical Growth and Development of Children (NSPGDC) in nine cities in China [17], which also showed declining trends in child stunting, underweight, and wasting for children under five years, and an increasing trend in the prevalence of overweight from 1985 to 2015. Similar results have also been observed in other countries. Between 1990 and 2015, the global rate of underweight and stunting was reduced by an estimated 42% and 41% [4]. Whereas, from 1990 to 2011, the global prevalence of child overweight under 5 years of age increased by 54% [1]. Continuously improved socioeconomical and educational conditions, especially maternal education, may be a major determinant for reducing childhood undernutrition. Higher purchasing power and nutrition awareness enables improved dietary intake in lower income families [18]. Child chronic undernutrition, such as stunting, and less improvement in maternal malnutrition, as well as poverty, may lead to a higher prevalence of child stunting. Maternal undernutrition and inadequate nutrition during pregnancy, infancy, and early childhood leads to stunting [5,19]. Child overweight and obesity is also related to economic progress. Excess energy intake in relation to energy expenditure leads to overweight and obesity [20]. Population changes in lifestyle behaviors are also risk factors for overweight, such as high levels of sedentary activity, low levels of child and parental physical activity, and high consumption of energy dense foods and beverages, such as sugary beverages [21,22].

In our study, the prevalence of EBF for infants aged 7–18 days and 0–5 months was extremely low, and remained unchanged in 1990 to 2016. In spite of the well-recognized importance of EBF, the practice is not widespread in many developing countries. Consistent with our study, the prevalence of EBF among infants under six months only increased from 33% to 39% in developing countries from 1995 to 2010, with the biggest improvement in Africa, while the East Asia and Pacific remained stable, at around 30%, during the past two decades [23].

Sex and regional disparities exist in child malnutrition. In present study, we found that the prevalence of child stunting, wasting, overweight, and obesity were higher among boys than girls in China, which is in accordance with the worldwide nutrition trends based on 2416 population-based measurement studies [24], as well as the studies in European [25,26], American [27], African [28,29], and Asian [30,31,32,33] countries. Consistent with results from other studies [34,35], we found girls have a higher prevalence of underweight in China. However, the NCD Risk Factor Collaboration revealed the prevalence of moderate and severe underweight in 2016 was higher in boys than in girls. Further, the prevalence of low birthweight was higher in girls than boys. Although similar sex disparities were observed in most countries, the explanations are unclear. One possible reason for this difference in China might be the social and cultural roles for boys and girls. Societal norms and parents may be more concerned about weight, thinness, and body size in girls than boys. Another reason may be that more resources, including food allocation favor boys [36,37], because of the son-preference in China.

We also observed regional differences in nutrition indicators. Child stunting, wasting, and underweight were highest in western China and lowest in eastern China, but child overweight and obesity showed opposite results. Interestingly, this finding was consistent with disposable personal income (DPI) in three regions. The average DPI in eastern, central and western China was 36,298.2, 23,798.3, and 21,935.8 CNY in 2018 [38]. Therefore, we speculate that parents/caregivers in central and western China may not have access or availability to a healthy and affordable diet and therefore pregnant women, infants, and children in western China have poorer nutrition status because of the lower economic development in these regions. In addition, pregnant women and infants in these two regions may lack nutritious foods and beverages, which may lead to poor maternal health and, in turn, malnourished infants and toddlers. On the contrary, children in higher DPI households are more likely to have access to and can afford healthier foods and beverages, as well as have more nutrition education; thereby, the prevalence of child undernutrition is lower in these regions. At the same time, the children in higher DPI regions, such as eastern China, face heavier academic pressures and may have more sedentary activity and use of electronic products, and also consume more energy dense, low-nutrition foods/beverages, and may be exposed to more marketing of unhealthy foods and beverages. These factors may be attributable to greater risk of overweight and obesity in eastern China. However, of concern, the increase overweight in western China has exceeded the central region since 2000. Therefore, action plans for curtailing rising rates of overweight and obesity need to be countrywide.

The Chinese government needs to shift priorities and concentrate greater efforts for children who face health problems of stunting, overweight, and EBF in order to meet nutrition-related SDGs and national targets. With the rural–urban migration and increasing urbanization process in China, more and more young parents and their children are migrating into cities, especially into eastern cities. However, the social and economic environment of migrants is worse than urban citizens. In addition, the social resources are limited for the cities migrants moving into, migrant children have fewer opportunities to receive high-quality health services and school education. Furthermore, the continuous migration from one city to another makes it difficult to track the nutritional status of migrant children. Therefore, we encourage the government keep the focus on reducing health inequities for the underserved population, establish specific scientific knowledge publicity programs, and allocate personnel to work on basic public health services for children and parents who have migrated. We may achieve the target SDGs for stunting if we can reduce the stunted number of migrated children in the eastern region.

Meanwhile, the gap between child overweight, infant EBF and meeting target goals is universal throughout China. Multi-sector and multi-component strategies are needed at the national, regional, and provincial levels, as well as collective efforts at the individual level, governmental level, and all sectors of society, because obesity is a complex and public health issue. We need to have government officials, NGOs, and the public, aware of the seriousness of childhood obesity and the rising rates and consequences that it will have for China and the health of the people. Additionally, if we focus solely on individual diet and physical activity behavioral changes without accompanying policies and programs to the change the larger food and built environment, we will not be able to prevent child obesity [39].

Several recommendations from WHO Report of the Commission on Ending Childhood Obesity can be applied in China. For obesity prevention and management, a life course approach needs to be considered, and specific interventions need to be implemented at each life phase [40]. During pregnancy, preconception weight and good antenatal care are essential for preventing excess weight gain during pregnancy. During infancy, breastfeeding and the timing of complementary foods are important. In childhood, parents and caregivers can role model a lifestyle of physical activity and healthy eating, and avoid energy dense highly processed foods high in sugars and fats. When children enter school, more changes in the food environment are needed, such as healthy meals and snacks, and plenty of physical activity. Having healthier foods in restaurants and calorie labeling in fast food chains is also needed. The government needs to regulate and restrict all marketing of unhealthy foods and beverages to children and adolescents and establish codes and nutrition standards for food advertising. Because it is well documented that sugary drinks, like soda, cause excess weight gain and obesity in children and adolescents, strategies to reduce sugary beverages are especially needed [41,42]. WHO recommends taxes on sugar-sweetened beverages as one strategy to reduce child obesity [43,44]. Mass media campaigns are also encouraged for obesity awareness and to promote a healthy eating and active lifestyle.

The same is true for efforts to increase EBF in the eastern region, which has the lowest prevalence of EBF. The promotion of EBF needs multiple efforts from individuals and collaboration across society. Besides improving the mother’s willingness to breastfeed [45] and educational level [46], breastfeeding peer counseling programs can effectively improve rates of breastfeeding initiation, duration, and exclusivity both in developing and developed countries [47]. Peer support interventions have been shown to increase breastfeeding by 30% and non-exclusive by 37% in low or middle income countries [48]. In addition, it is critical to enforce the WHO Code for Marketing of Breastmilk Substitutes, as well as the successful large-scale implementation and sustainability of the Baby Friendly Hospital Initiative [49].

This study presented many strengths, such as the first combination of two large database, the scientific predictive model, multi-level analysis (gender and region), and all the child nutrition indicators from SDGs. There are also limitations to consider. Given that GBD data were more internationally comparable, we mainly used GBD 2016 data in our analysis. However, the GBD 2016 have been increasingly model-dependent in recent years and limitations regarding data gaps, methodological challenges are described elsewhere [50]. We could not examine the urban-rural disparities in child malnutrition with GBD data. Additionally, the socio-demographics of child nutrition were not included in our data sets, so we cannot reasonably explain the changes and differences in child nutrition at the socioeconomic level. Further, the projection methodology can only reflect future changes based on historical trajectories, and demographic and socioeconomic factors could not be added into the model. Furthermore, other possible changes, such as political commitment and technology advances and interventions, were not considered in the projections.

## 5. Conclusions

Chinese children’s nutritional status shows a trend from malnutrition to overweight and obesity in the past 26 years. Undernutrition in children under five years in China has remarkably improved, although the prevalence of child stunting has decreased it remains high at 12%. Child overweight and obesity is progressively increasing, and the prevalence of overweight has doubled and obesity has tripled in the past few decades. Exclusive breastfeeding has not increased from 1990 to 2016 and it remains low, at 30%. Sex and regional nutrition and weight disparities exist: the prevalence of child stunting, wasting, overweight, and obesity are higher among boys than girls, child undernutrition is higher in western China, and prevalence of child overweight and obesity is higher in eastern China. Our projected results highlight the urgent need for national and regional policies and multi-level interventions to increase EBF and reduce child stunting and overweight/obesity. Targeted interventions are needed since the prevalence of child stunting, wasting, overweight, and obesity are the highest among boys. For obesity targeted interventions are needed for children in eastern China. If China can improve nutritional health by universal access to a healthy and affordable diet needed for healthy growth and weight for all pregnant women and infants and children and also improve infants’ exclusive breastfeeding, Chinese children will have a healthier future, and China will have a stronger country and future workforce and will achieve a historic public health leap.

## Figures and Tables

**Table 1:** Child nutrition trends from 1990 to 2016 in China ^a^.

	1990	1995	2000	2005	2010	2016
Stunting ^b^ (%)	28.1	26.0	19.9	15.1	13.4	11.6
Wasting ^b^ (%)	5.8	5.5	4.0	3.2	2.9	2.7
Underweight ^b^ (%)	12.0	10.4	7.5	5.1	4.5	3.7
Overweight ^c^ (%)	6.3	8.2	9.5	9.4	10.1	11.9
Obesity ^c^ (%)	2.2	2.7	3.9	4.4	5.3	6.9
EBF ^d^ (%)	30.0	30.7	31.8	33.0	32.1	30.2

^a^ Data source: Global Burden of Disease (GBD) 2016; ^b^ Children under 5 years, ^c^ Children aged 1–4 years, ^d^ Exclusive breastfeeding for infants aged 7–28 days.

**Table 2:** Gap analysis results for nutrition indicators in China.

Indicators	Status (2016)	Target Value	Predictive Value					
	National Level	National Level	National Level	Boys	Girls	Eastern China	Central China	Western China
Stunting ^a^ (NO. in 10 000)	704.3	422.6 ^#^	424.8	250.5	175.3	165.8	149.5	113.3
Wasting ^a^ (%)	2.7	5.0 ^#^	2.0	2.3	1.7	1.5	2.4	2.4
Overweight ^b^ (%)	11.9	11.9 ^#^	14.1	16.3	11.7	17.0	11.7	11.5
EBF ^c^ (%)	30.2	50.0 ^#^	30.3	30.3	30.3	26.9	33.2	33.7
Underweight ^a^ (%)	3.7	5.0 *	3.1	3.0	3.2	2.2	3.7	4.2
Stunting ^a^ (%)	11.6	7.0 *	8.3	9.0	7.4	6.7	9.9	9.7
	11.6	5.0 *	6.8	7.5	6.0	5.6	8.3	8.0
Low birthweight (%)	3.3	4.0 *	—	—	—	—	—	—

^a^ Children under 5 years, ^b^ Children aged 1–4 years, ^c^ Infants aged 7–28 days. ^#^ Targets set by Sustainable Development Goals (SDGs). * Targets set by Chinese government.

## Supplementary Materials

The following are available online at https://www.mdpi.com/1660-4601/17/4/1129/s1, File S1: Introduction of Trend Calculator; Figure S1. Undernutrition for children under 5years in 3 regions in China (%); Figure S2. Undernutrition for children under 5 years in China by gender (%); Figure S3. Overweight and obesity for children aged 1–4 in 3 regions in China (%); Figure S4. Overweight and obesity for children aged 1–4 in China by gender (%); Figure S5. EBF for newborns aged 7–28 days in 3 regions in China (%).
