# Supplementary material for: Child Nutrition Trends Over the Past Two Decades and Challenges for Achieving Nutrition SDGs and National Targets in China
Source: Int J Environ Res Public Health. 2020 Feb 11;17(4):1129. doi: 10.3390/ijerph17041129 (PMC7068302; doi:10.3390/ijerph17041129)
Supplement: Supplementary file 1 [file ijerph-17-01129-s001.pdf]

Supplementary Materials

(1) Introduction of Trend Calculator

Trend Calculator following the original methodology to first convert data into logit-space (for percentage data) or natural-log space (for other data) and calculate the annual rate of change. Afterwards, a time-based weight matrix was established to add more weight to rate of change in recent years. The weight of rate of change in a selected year is:

$$Weight_{year} = \frac{(year - 1990)^{\omega}}{\sum_{t=1991}^{T}(t - 1990)^{\omega}}$$

Where T is the last year with available data. The parameter ω was determined with a validity test: we selected the ω that used the first half of available data to predict the second half most accurately. Building upon the weight matrix, we calculated the weighted mean annual rate of change and used it to predict the data trend.

Trend Calculator can weaken the influence of data fluctuations by taking the natural logarithm or logit method in advance, and avoid the reasonable limit of the percentage data exceeding (0,100). At the same time, this method used the previous part of the original data for trial operation and establish the weight matrix based on the year of the data. Three data points were needed at least for the method, and 5 data points or above can predict the average value by the weight of the year, that was the reason why we did not project the low birthweight indicator.

(2) Figures about child malnutrition over the past two decades

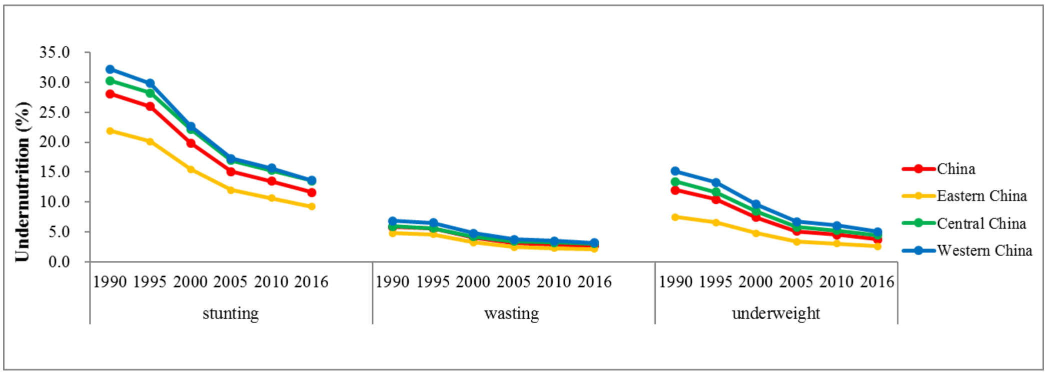

Figure S1. Undernutrition for children under 5 years in 3 regions in China.

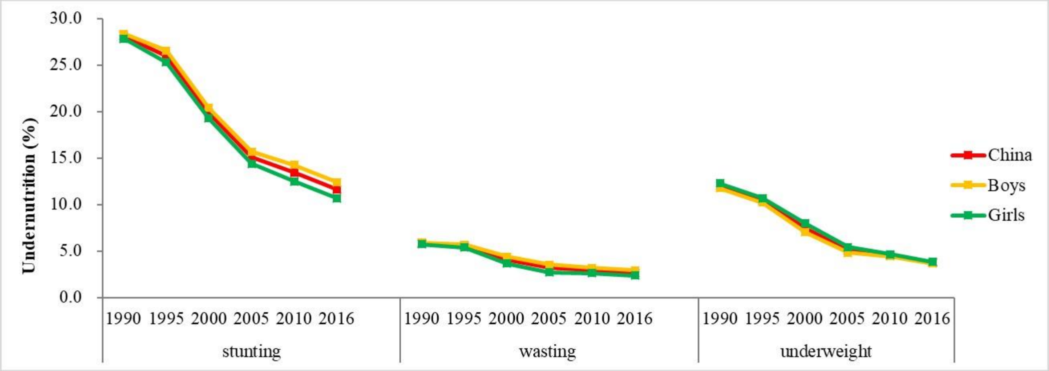

Figure S2. Undernutrition for children under 5 years in China by gender.

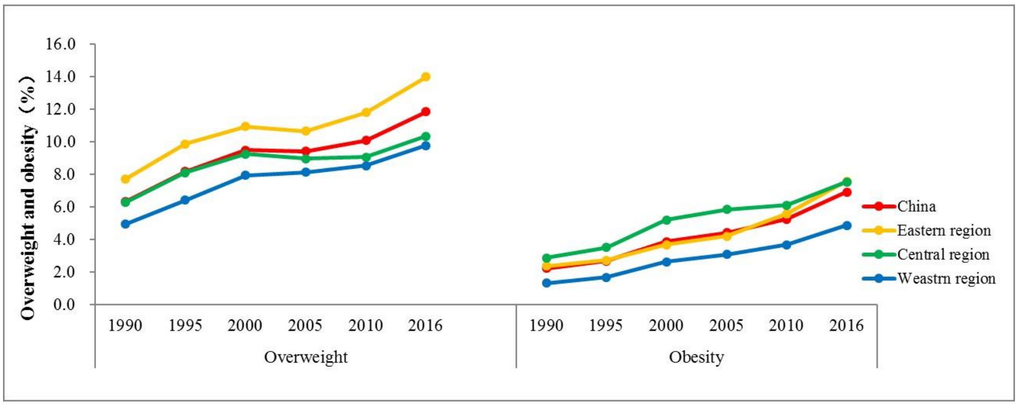

Figure S3. Overweight and obesity for children aged 1-4 in 3 regions in China.

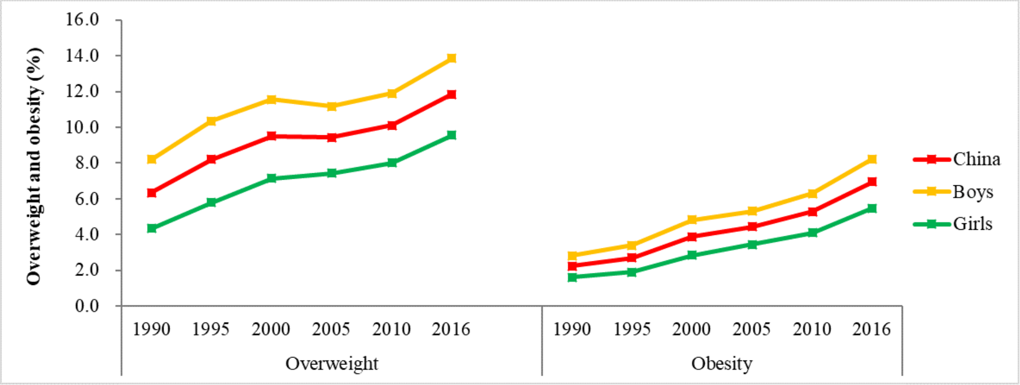

Figure S4. Overweight and obesity for children aged 1–4 in China by gender.

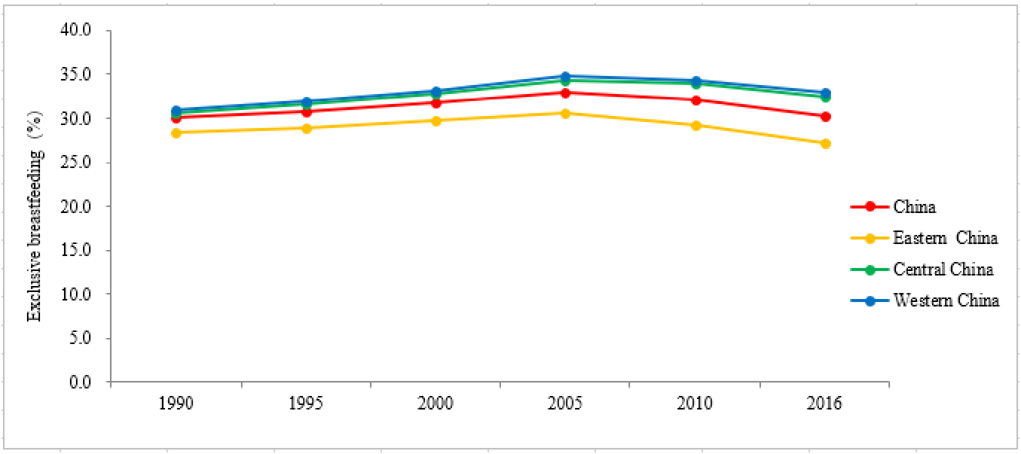

Figure S5. EBF for newborns aged 7–28 days in 3 regions in China.
